# Investigating the acceptability of cervical screening, using conventional clinician-taken cervical samples or urine self-sampling, at 6 weeks postnatal: A cross-sectional questionnaire

**DOI:** 10.1177/09691413251358626

**Published:** 2025-07-21

**Authors:** Victoria Cullimore, Rebecca Newhouse, Holly Baker-Rand, Kim Chu, Sudha Sundar, Emma J Crosbie, Lorna McWilliams, Jo Morrison

**Affiliations:** 1 Department of Gynaecological Oncology, 7852 Somerset NHS Foundation Trust, Taunton, UK; 2 Faculty of Health and Life Sciences, 3286 University of Exeter, Exeter, UK; 3 Department of Gynaecological Oncology, 7425 University Hospital Southampton NHS Foundation Trust, Southampton, UK; 4 Cancer Prevention & Early Detection Research, NIHR Manchester Biomedical Research Centre, 5292 University of Manchester, St Mary's Hospital, Manchester, UK; 5 Wolfson Institute of Population Health, 4617 Queen Mary University of London, London, UK; 6 Cancer Sciences, 1724 University of Birmingham, Birmingham, UK; 7 Pan Birmingham Gynaecological Cancer Centre, Midlands Metropolitan University Hospital, Birmingham, UK; 8 Manchester Centre for Health Psychology, School of Health Sciences, University of Manchester, Manchester, UK

**Keywords:** Cervical screening, postnatal, pregnancy, HPV testing, self-sampling, mixed methods, acceptability

## Abstract

**Objectives:**

United Kingdom (UK) guidelines recommend delaying cervical screening due during pregnancy to 12 weeks postnatal, despite a lack of supporting evidence. This questionnaire-based study aimed to determine the feasibility of a clinical study of cervical screening and urine self-sampling for human papillomavirus (HPV) at 6 weeks postnatal, as pilot work suggested this would improve uptake, if offered at the routine postnatal check-up.

**Methods:**

Females who were pregnant/recently pregnant were invited to participate in a web-based questionnaire. Questions assessed acceptability of postnatal cervical screening at 6 weeks postnatal, analysed with chi-square, Fisher's exact and Mann–Whitney tests. Free-text responses were coded using the Theoretical Framework of Acceptability (TFA) to conduct a qualitative content analysis.

**Results:**

Among the 454 participants, 266 (58.6%) would be more likely to undergo cervical screening if offered at 6 weeks postnatal, and an even higher proportion expressed increased willingness if urine self-sampling were offered (*n* = 338; 74.4%). Two-thirds (308/454; 67.8%) would be willing to be screened at 6 weeks postnatal for a research study and 356/454 (78.4%) if it would be limited only to urine self-sampling. When considering screening modality, over half (245/454; 54%) would prefer urine self-sampling to cervical screening, although a fifth (93/454; 21%) preferred conventional sampling. Free-text responses were provided by 279 participants, and these highlighted that affective attitude and burden TFA constructs underpinned prospective acceptability of having screening at 6 weeks postnatal.

**Conclusions:**

Offering cervical screening at the 6-week postnatal check-up has potential to increase cervical screening participation. Most participants would be interested in taking part in the research. The feasibility of screening at 6 weeks postnatal and concurrent acceptability should be tested in pilot clinical studies.

## Background

Cervical cancer is one of the most preventable malignancies worldwide.^1^ The National Health Service Cervical Screening Programme (NHS CSP) began in 1988, initially using conventional Papanicolaou ‘smears’, later replaced by liquid-based cytology (LBC).^2^ LBC was subsequently augmented with high-risk human papilloma virus (hrHPV) triage of low-grade cytological abnormalities and, more recently, primary hrHPV screening.^3–5^

Since its introduction, studies show that NHS CSP has reduced cervical cancer mortality by 70%.^6^ However, the mortality benefit requires widespread population uptake, with a target of 80% in the UK.^7^ By 2022/23, cervical screening coverage rates in England were at an all-time low, at around 67% in the under 50s, and well below 40% in some areas.^8–10^ Other studies have found that rates are especially low in younger people with a cervical cancer and those with children aged less than 5 years.^9,11^ A disproportionate number of cervical cancer cases occur in those who do not attend for screening and, importantly, this is increasing.^12,13^ Screening rates are particularly low in certain groups, including ethnic minorities, those with higher index of deprivation, and those with young children.^11,14–16^ There are many barriers to cervical cancer screening, including perception that young people are not at risk, practical barriers that hinder attendance, inadequate knowledge, and fear of pain, discomfort and embarrassment.^17,18^ A recent systematic review identified being ‘too busy’ and childcare constraints as significant barriers to screening attendance among young women.^19^

The peak age incidence of cervical cancer in the UK, between 2016 and 2019, was in the 30–34-year age group, followed by those aged 25–29 years.^13^ Within the 25–34-year age group, the proportion of those diagnosed with cervical cancer in those never-screened increased from 39% to 58% in a 10-year period.^13^ There is a clear need for research to increase screening uptake in younger women.^20^ The average age of mothers giving birth in England and Wales is 30.9 years, corresponding to the peak incidence of cervical cancer.^21^ In the UK, those with high index of multiple deprivation are more likely to be younger at maternity,^22^ although the 30–35-year age group is the most common age for maternity across all ethnic groups.^23^ Pregnancy provides several points of contact to engage women in health promotion and therefore opportunities to educate and screen, especially in underserved groups.^21,24^ Previous work found that, by the end of pregnancy, nearly half were out-of-date with their cervical screening, and 74% of this group had still not had screening by 6 months postnatal.^25^ High parity is associated with an increased risk of cervical cancer.^26^

The national guidance currently advises waiting 12 weeks after childbirth for routine cervical screening, if the previous result was normal and the test was due in pregnancy.^27^ Cervical samples collected during pregnancy can be challenging to interpret by cytology,^28^ although there are few data suggesting that hrHPV-based postnatal cervical screening should be delayed until 12 weeks postnatal. One randomised comparison of postnatal Papanicolaou smears, taken at 4, 6 and 8 weeks postnatal,^29^ prior to the introduction of LBC and hrHPV testing, found increased inflammatory changes in samples collected closer to delivery. However, the most challenging were samples taken at 4 weeks when, of those with normal prenatal screening, 26/44 had abnormal cytology (24 due to inflammatory changes (55%): 2 dyskaryosis), and there was little difference between those at 6 weeks (16/53: 15 due to inflammatory changes (28%); 1 dyskaryosis) and 8 weeks postnatal (12/42: 11 due to inflammatory changes (26%); 1 dyskaryosis). They were unable to compare the early postnatal screening with repeat screening 3 months later due to high rates of loss to follow-up. LBC improves the adequacy rates of cervical screening and reduces the number of falsely abnormal screening tests due to inflammatory cells.^30–32^ An observational study reported no difference in inadequate LBC rates (2.5%; *n* ∼ 800 women) when the smear was taken at 6 weeks postnatal, compared to 2.7% in a non-pregnant gynaecological population (*n* = 1429).^33^ Using LBC likely negates the previously held belief that postnatal smears should be performed after 12 weeks, although data are weak and underpowered. Furthermore, this study pre-dated hrHPV testing, and 6- and 12-week screening were not directly compared; the inadequacy rates of hrHPV primary testing are even lower (<1% in under 40s). Current recommendations are therefore based on very low certainty data using out-dated tests and may inadvertently contribute to lower screening rates in this important patient cohort.

The National Institute for Health and Care Excellence (NICE) recommends a 6-week postnatal check-up for all mothers and babies, which is currently attended by 78% of postnatal women.^34,35^ This provides an opportunity for healthcare professionals to discuss multiple topics, including breastfeeding, lifestyle advice, and contraception.^36^ Combining cervical screening with the 6-week postnatal check-up was suggested by primary care staff and mothers of young children in our previous quality improvement study.^25^

The objectives of this cross-sectional study were (a) to understand how willing new mothers to participate in a clinical trial comparing different postnatal testing strategies, including urine self-sampling, and (b) the acceptability of having a cervical sample taken and of self-sampling for postnatal women at the 6-week postnatal check, compared to waiting until 12 weeks. This is in line with the guidance from the Medical Research Council on the development and evaluation of complex interventions.^37^ These data form the first step in a multi-stage feasibility study, including comparison of accuracy of testing at 6 versus 12 weeks, with the overall aim of addressing the evidence gap in postnatal cervical screening and assessing possible solutions to target this under-screened population.

## Methods

### Design

Participants were recruited from across England via posters displayed in primary and secondary care facilities via clinical research networks at multiple sites across England (e.g. general practitioner (GP) practices, antenatal clinics, and colposcopy clinics), and as advertisements within patient-held electronic maternity care records (Badgernet), the NIHR website (//bepartofresearch.nihr.ac.uk/), community-based children's centres and organisations, and via social media (Mumsnet, Maternity Voices, Facebook groups). Participants could access the online participant information sheet (see online Supplemental Material: questionnaire) and contact the study team with any questions prior to consent. The planned sample size was >100 participants, and data collection continued until saturation of themes was achieved within free-text responses.

Ethical approval was obtained [Health Research Authority and Health and Care Research Wales – IRAS ID 321358/ Research Ethics Committee (REC) reference 23/SC/0082], and participants provided informed consent online prior to completing the questionnaire. The study was pre-registered on the NIHR website (//bepartofresearch.nihr.ac.uk/); CPMS ID: 55489; clinical trial registration was not applicable.

### Participants

Inclusion criteria included anyone who was eligible for the CSP (female, aged between 24.5 and 65 years, still had a cervix) and was either pregnant or had been pregnant within the past 5 years.

### Measures

The primary objective of this study was to assess the feasibility of a research study to assess cervical screening at 6 weeks postnatal. Participants were asked about their willingness to take part in a cervical screening research study at 6 weeks postnatal, with a yes or no response. This was asked in two separate questions: (a) if cervical screening were performed with conventional screening methods (clinician-taken cervical sample) or (b) by urine self-sampling.

The web-based questionnaire (Microsoft Teams; see online Supplemental Material: questionnaire) included questions about sociodemographic characteristics (ethnicity, educational attainment, age in 10-year brackets, parity), method of transport to hospital (flagged by patient and public involvement and engagement groups as a potential barrier to a hospital-based study), and cervical screening status, including reasons for not attending or delaying screening.

To test the acceptability of offering cervical screening alongside the 6-week postnatal check-up, we asked four questions to assess acceptability and how this might influence participants’ future screening uptake. Participants were asked to rank on 5-point Likert scales whether they would be more likely to have cervical screening if offered at the 6-week postnatal check-up, with either conventional screening or urine self-sampling, whether they would prefer urine self-sampling generally, and whether they would prefer to have screening at 6–12 weeks postnatal. Participants were finally asked to share any thoughts on cervical screening, types of tests, or screening after delivery in a free-text box with no word limit. All responses were anonymous, but participants had the option to provide their email address, if they would be willing to have a 1-h interview in a follow-on qualitative study (findings will be reported elsewhere) to explore their views in greater depth.

### Analysis

#### Quantitative analysis

Post hoc exploratory analysis evaluated independence between categorical variables using chi-square or Fisher's exact tests; and the Mann–Whitney test is used to compare two independent quantitative variables. Statistical analysis was performed with Microsoft Excel^38^ and GraphPad Prism software.^39^

#### Qualitative analysis

All data within free-text responses were analysed using qualitative content analysis to further explore prospective acceptability.^40^ A deductive codebook was developed (see online Supplemental Material: codebook), and data were entered into Excel using each of the seven constructs within the Theoretical Framework of Acceptability (TFA): affective attitude, burden, ethicality, perceived effectiveness, opportunity cost, intervention coherence, and self-efficacy.^41,42^ The TFA, developed by Sekhon et al., was designed to facilitate research that focused on evaluating acceptability of healthcare interventions prospectively, concurrently, or retrospectively.^41,42^ Each construct had three codes based on whether the data extracts related to speculum-based screening, timing of screen (i.e. 6 weeks postnatal), or self-sampling testing. Following pilot coding of the first 50 participants, we agreed that opportunity cost was not a helpful construct, as participants were considering hypothetical acceptability. Additional columns were added beside each construct code to indicate whether the research team interpreted the data extract as a positive, negative, or neutral opinion, where relevant. A third column allocated each data extract to categories from a list that was iteratively developed throughout data coding consisting of psychological birth trauma, physical birth trauma, birth trauma (not specified), practicality (baby at appointment, multiple appointments, appointment availability, or other), cancer prevention/detection, maternal health prioritisation, embarrassment, fear, bleeding post-delivery, invasiveness, accuracy, pain/discomfort, and recovery from birth. Extracts may have been allocated to multiple categories.

Three members of the research team (VC, LMcW, JM) independently coded all free-text responses, with each being analysed by a minimum of two researchers. Coding was compared and there was agreement by consensus between two coders (VC and LMcW), and with a third (JM) if required. Data were then analysed together according to construct, sub-topic, and direction, to better understand the common findings. Every aspect of each comment was initially coded; however, only data relevant to the research questions were included in the reported findings. The categories and constructs were first used to contextualise the relevant quantitative findings regardless of specific TFA construct relating to the acceptability of speculum-based screening, cervical screening offered 6 weeks postpartum, and urine as a cervical screening test. Then, the categories used across the TFA constructs were used as the thematic structure to present what participants shared in relation to speculum-based screening, timing of screening, and urine for self-sampling. As researchers, we regularly engaged in reflexivity, noting how our different professional backgrounds and personal experiences could influence interpretation. Factors we considered are as follows: (a) all three of the authors involved in qualitative analysis were female with personal experience of cervical screening; (b) at least one had personal experience of treatment for abnormal cervical screening; (c) none of the three had given birth, although two had considerable experience on the other side (as obstetricians) and over half of the author team did have personal experience of pregnancy; and (d) two had experience of seeing and treating young pregnant women and new mothers with cervical cancer.

## Results

### Participant characteristics

Between April 2023 and May 2024, there were 454 participant responses (one test response by researcher excluded). Of those who read the electronic patient information sheets, 454 (100%) consented to the study, with no opt outs, and 454 (100%) provided demographic details at the end of the survey. Of those, 273 (60%) entered a response in the free-text box. The median age cohort was 30–34 years, and the median number of children was two (range in the first pregnancy to more than six children) (Table 1). The majority described themselves as White (*n* = 407; 89.6%) and thus, for the purposes of analysis participants, were recorded into the broad categories of White or non-White. Fifty-four participants (11.9%) were currently pregnant, and the majority were either pregnant or had had their most recent pregnancy within the last 2–3 years (82.2%). A high percentage of participants had a university or post-graduate degree (*n* = 379; 83.5%); 409 participants (90%) were employed, 4 (0.9%) were students, 37 (8%) were homemakers, and only 4 (0.9%) were unemployed.

**Table 1.** Characteristics of study participants.

	Number (total = 454)	Percentage
**Age**		
24–29 years old	82	18.1%
30–34 years old	191	42.1%
35–39 years old	135	29.7%
40+ years old	46	10.1%
**Education**		
Up to higher or secondary or further education (A Levels, BTEC, etc.)	75	16.5%
University or college	232	51.1%
Post-graduate degree	147	32.4%
**Number of children**		
0	28	6.2%
1	177	39.0%
2	184	40.5%
3+	65	14.3%
**Last cervical screen (self-reported)**		
Never-screened	19	4.2%
Over 3 years ago	73	16.1%
Within the past 3 years	362	79.7%
**Mode of transport**		
Car (self/family/friend)	397	87.4%
Public transport including taxi	26	5.7%
Walk/cycle	31	6.8%
**Ethnicity**		
White	407	89.6%
Non-White	47	10.4%
**Birth of last child**		
Currently pregnant	54	11.9%
Within the past year	66	14.5%
1–2 years ago	86	18.9%
2–3 years ago	167	36.8%
3+ years ago	81	17.8%

**Figure 1.**
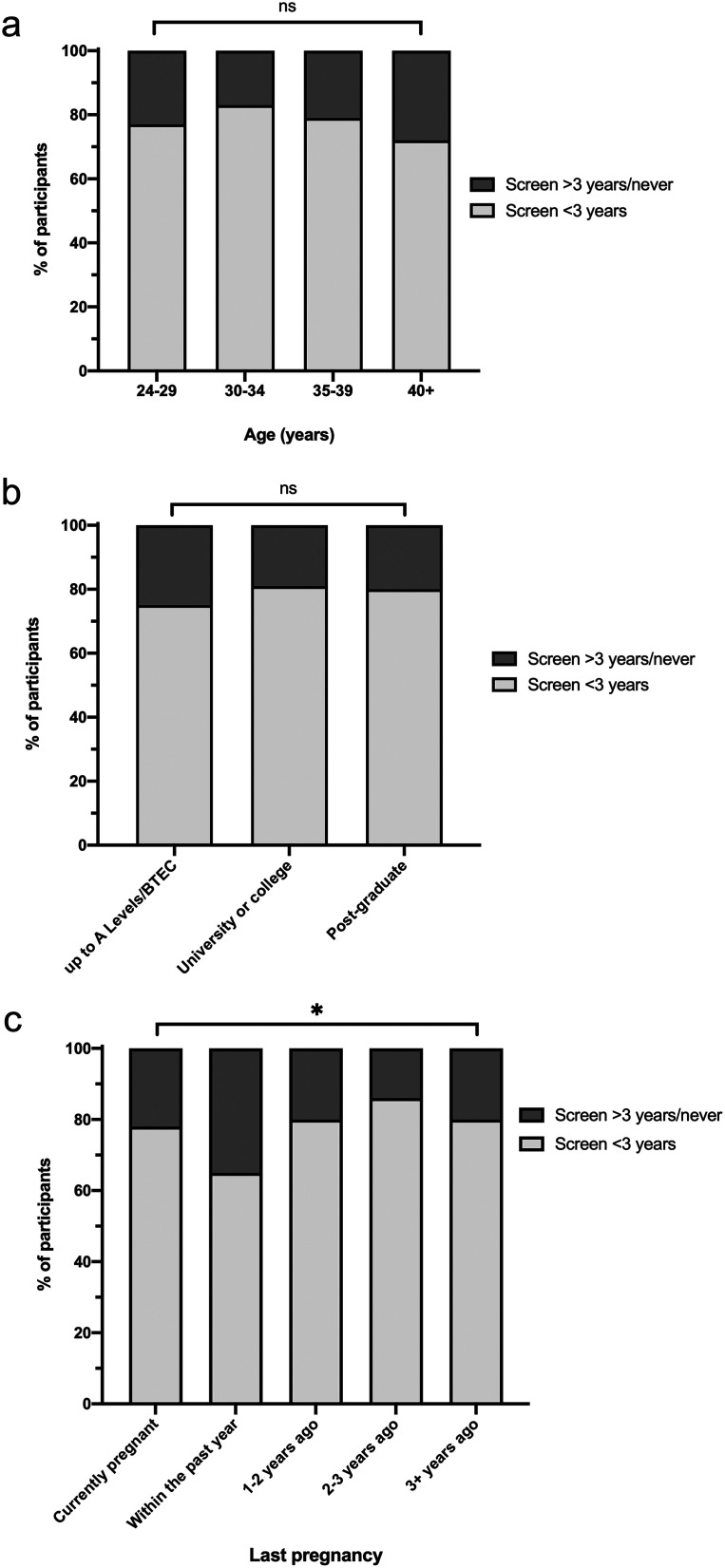
Associations between cervical screening status and (a) age, (b) educational status, and (c) recency of last pregnancy. Those with self-reported cervical screening tests within the last 3 years are shown in light grey and over 3 years ago (including never) in dark grey. ns = not significant; *p = 0.014. Data shown as % of each category; *n* = 454 in total for each graph. See text for further details.

The age distribution of participants was similar by time since the last screen; 362 women reported screening within 3 years (79.7%), 73 screened >3 years ago (16.1%), and only 19 were never-screened (4.2%) (Figure 1(a); χ^2^ (3) = 3.72; *p* = 0.29). Educational attainment was also similar by screening status (Figure 1(b); χ^2^ (2) = 1.46; *p* = 0.48). However, those whose last pregnancy had been longer ago were slightly more likely to be up to date for their screening (Figure 1(c); χ^2^ (4) = 12.43; *p* = 0.014).

### Willingness to take part in a 6-week postnatal cervical screening study

Two-thirds of participants (*n* = 308; 67.8%) stated they would be willing to take part in a clinical trial involving cervical screening at 6 weeks postnatal, and this proportion of participants increased to over three-quarters (*n* = 356; 78.4%) if urine self-sampling were the only option. Interestingly, 302/308 participants who were willing to take part in a cervical screening study at 6 weeks postnatal were also willing to take part in a urine self-sampling study. However, those who were not willing to take part in a cervical screening study at 6 weeks postnatal were much less likely to be willing to take part in a urine self-sampling study (92/146; 63.0%; *p* < 0.0001; Fisher's exact test):

P203: *6 weeks after birth I still don’t know what day of the week it is or what my name is. I’m trying to focus my whole life on a new life I have to care for, so the absolute last thing is to add another thing to my to do list and to worry whilst waiting for the results!* (burden)

There was greater willingness among those currently pregnant (42/54 78%) or within a year of their last pregnancy (51/66: 77%) to take part in a cervical screening study than those whose pregnancy was over a year ago (215/334; 64%) (χ2 (1) = 6.12; *p* = 0.013; test for trend). This perhaps reflects an increased likelihood of giving birth in the future, so less of a hypothetical question, although reasons were not sought. Willingness to take part in a cervical screening study was not significantly related to screening status (never-screened = 11/19 (58%); >3 years = 55/73 (75%); within 3 years = 242/362 (67%); χ2 (1) = 0.11; *p* = 0.74; test for trend).

When asked if they would prefer to have cervical screening at 6 weeks rather than 12 weeks postnatal, participants’ responses were relatively balanced, with 30.8% (*n* = 140) ‘disagreeing’ or ‘strongly disagreeing’ with a 6-week screen and 30.6% (*n* = 139) ‘agreeing’ or ‘strongly agreeing’. Interestingly, 39% (*n* = 175) of participants neither agreed nor disagreed, and comments in the free-text analysis demonstrated that answers to this question are more nuanced than yes or no. Participants commented that their decision would be dependent on their childbirth experience (*n* = 36) and recovery (*n* = 30). For example:

P325: *If it had been 6 weeks after my daughter's delivery, I would have been fine with it. She was an uncomplicated delivery. My son, who was born with forceps, left me in considerable pain for months and there’s no way I would have wanted a smear test 6 weeks after giving birth, I was nowhere near healed enough. So, it's really dependent on the woman*. (burden)

P282: *[Cervical screening is] Very uncomfortable and quite triggering after a traumatic birth, with very little support available.* (affective attitude)

Over half of participants (*n* = 266; 58.6%) agreed or strongly agreed that they would be more likely to have postnatal cervical screening if offered at the time of their postnatal check-up, with 119 disagreeing or strongly disagreeing (26.2%) and 69 (15%) neither agreeing nor disagreeing. Participants felt that scheduling cervical screening at the 6-week postnatal check-up would be more convenient, as it would combine both appointments into one, making it easier to manage time with a newborn. This was reflected in 20 comments highlighting that this could avoid the need for multiple appointments during a physically and cognitively demanding time:

P11: *Would be more likely to attend if the appointments were linked with others… The first few weeks after delivery, I would never remember to organise something for myself like that*. (burden)

P200: *I can see that it would be more difficult to go to an appointment just for [yourself] with a newborn baby and think I’d be more likely to have a test if the appointment is combined with a check of my baby.* (burden)

On the other hand, 72 participants highlighted drawbacks of earlier postnatal screening, most commonly citing concerns about birth trauma, the pain or discomfort associated with screening, and the challenges of recovery from childbirth:

P187 *I didn’t even have a vaginal birth but the thought of a smear test so soon after pregnancy terrifies me.* (affective attitude)

P143: *I think post-delivery cervical screening is a good idea, but I’d be worried that I’d still not be totally healed after 6 weeks so a smear would hurt more than usual, compared to longer after delivery.* (burden)

Three-quarters of the participants (*n* = 338; 74.4%) reported that they would be more likely to have 6-week postnatal cervical screening if it was by urine self-sampling, 81 (17.8%) neither agreed nor disagreed, and only 35 (7.7%) women felt they would disagree or strongly disagree to this offer. This is illustrated by the following participant describing their fear towards a cervical screening test at 6 weeks whilst feeling positively about a urine test at the same time point:

P115: *Screening after delivery scares me. I’ve delayed mine as a result. I have it coming up and even after 16 weeks post delivery, I’m terrified. A urine sample test would be amazing to me right now! Pre-pregnancy smear tests never bothered me.* (affective attitude)

### Self-sampling preferences

**Figure 2.**
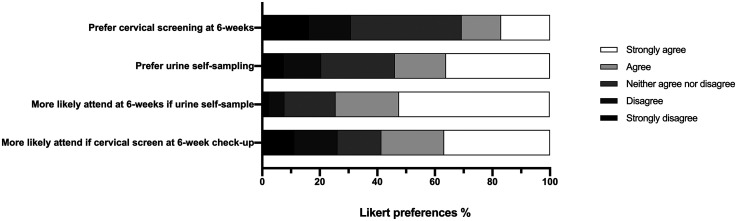
Preferences for different screening options. Respondents were asked to rate their agreement with statements on a 5-point Likert scale from strongly disagree (black) to strongly agree (white). Statements for agreement/disagreement were as follows: ‘I would be more likely to have my cervical smear after pregnancy if it was at the time of my 6-week postnatal check’; ‘I would be more likely to have cervical screening 6 weeks after delivery if I only had to provide a urine sample for testing’; ‘I would prefer to do self-testing by providing a urine sample than have a smear test’; and ‘I would prefer to have cervical screening at 6 weeks post-delivery than at 12 weeks post-delivery’.

Over half of all participants agreed/strongly agreed that they would prefer urine self-sampling (*n* = 245; 54.0%) to conventional cervical screening, whilst one-fifth preferred conventional screening (*n* = 93; 20.5%) (Figure 2). Free-text comment analysis demonstrated that self-sampling was preferred to speculum-based screening primarily because it is less invasive, particularly when considering the 6- or 12-week postnatal time point (see Table 2). At these time points, the option of self-sampling was described very positively as ‘amazing’ (participants (P) 115, 122, 422) and ‘excellent’ (P327), and participants said they would be ‘very happy to provide [urine]’ (P213). However, 15 participants expressed concerns about the accuracy of self-sampling for cervical screening, with respondents questioning its reliability or raising worries about their ability to perform the test correctly:

P305: *The urine test sounds easier, especially if self-sampling, but I don’t know how accurate the urine version of the test is. I’d prefer to take whichever test is better at picking up cancer.* (perceived effectiveness)

Others were more in favour of a vaginal examination following childbirth, to check everything had healed well, and several comments (nine participants) focussed on what was perceived lack of maternal health prioritisation at the postnatal check-up visit:

P95: *I would have loved someone to examine me at [postnatal] check after my first (difficult) delivery, and so by doing a smear at [postnatal] check I’d have felt at least someone checked things were okay.* (perceived effectiveness)

**Table 2.** Content analysis of free-text comments using the theoretical framework of acceptability, with example comments.

		Direction of quote						
		Positive		Neutral		Negative		No direction*
**TFA construct and aspect of research aims**		**Number of responses**	**Example quote**	**Number of responses**	**Example quote**	**Number of responses**	**Example quote**	
**Affective attitude**	Speculum-based screening	8	P54: ‘I’m comfortable having the traditional smear tests’	3	P316: ‘I don't mind having a smear’	13	P276: ‘It's incredibly invasive and unfortunately staff I’ve encountered have acted like letting a stranger put things in my vagina shouldn't bother me at all’	
	Time of screen	17	P200: ‘I’m happy to have cervical screening at any point’	10	P285: ‘Happy to have smear test at 6 or 12 weeks post-delivery – just depends if able to have your baby there with you’	50	P61: ‘After the type of delivery I had (forceps) which left with me with traumatic birth injuries (high apex tear), I would have been horrified if my GP suggested a smear test at 6 weeks postpartum’	
	Self-sampling	21	P6: ‘I’d be happy to do a urine-based test at any time postpartum (or otherwise)’	0		1	P249: ‘I would personally not be happy with a smear test that just involved a urine sample. I do not feel it is a thorough enough test’	
**Burden**	Speculum-based	1	P219: ‘I think they are fine, as a woman if you’ve had a coil inserted. Or even had your vagina waxed its nothing worse than that	6	P164: ‘Whilst uncomfortable, I have never not attended for a screening appt’	24	P160: ‘For those who have been through trauma such as sexual assault, having a cervical screening can be massively triggering’	
	Time of screen	37	P45: ‘It would be more convenient at the 6-week check-up as I already have my baby with me and have to attend rather than an additional appointment’	31	P91: ‘It depends on how the birth was. My first birth caused a lot of trauma’	70	P202: ‘Smear tests are necessary, but the trauma from birth has put me off wanting mine so close to delivery’	
	Self-sampling	11	P170: ‘Urine test would be much nicer and less invasive/painful. I would opt for this over a tradition smear test’	3	P305: ‘The urine test sounds easier especially if self-sampling, but I don’t know how accurate the urine version of the test is. I’d prefer to take whichever test is better at picking up cancer’	1	P285: ‘Prefer not to do self-test in case of error/you’d still have to deliver it so not necessarily more convenient’	
**Ethicality**	Speculum-based	41	P10: ‘I think cervical screening is very important; I attend all of my smears and encourage those around me to do so as well’	12	P204: ‘The age for smear tests needs to be lowered’	4	P276: ‘Let's be honest, of it were men with the cervix we wouldn’t still be testing in this way’	
	Time of screen	11	P302: ‘I personally wouldn’t find any preference but appreciate it's definitely more convenient when already having s check up and would also help to encourage more discussion around women's health and wellbeing postnatal as opposed to just the baby at the six week check’	3	P97: ‘Screening after delivery sounds daunting – especially if there are stitches. However it could serve a dual purpose as currently the 6-week postnatal check is woefully inadequate for women and if a cervical screening could also monitor post-birth healing and pick up on issues at an earlier stage could be really beneficial to women’	4	P61: ‘I think I would have felt pressured to agree to it in fear of wasting the doctor's time’	
	Self-sampling	6	P15: ‘More people are likely to get tested if self-testing available’	1	P52: ‘All types of test and options should be publicised and made clear to all’	0		
**Perceived effectiveness**	Speculum-based	6	P7: ‘It is important for anybody who has a cervix to take up cervical screening in a timely manner when offered, as this could be a lifesaving intervention’	0		1	P187: ‘I also worry that just testing for HPV is not enough’	
	Time of screen	3	P70: ‘I would have been nervous to have had the test 6 weeks after delivery as it is so soon after birth, but would have happily have had it done if it's proven to be just as effective and no more painful’	3		3	P310: ‘There are so many changes and hormonal shifts post-birth. It doesn’t seem as though it would be a good time to get an accurate test result’	
	Self-sampling	0		11	P47: ‘It would be useful to have clarification on if the urine test is as thorough/accurate as the smear test as if not that would put me off the urine test’	6	P29: ‘I would rather have a cervical smear as to me I would feel that's more reliable than a urine test’	
**Intervention coherence**	Speculum-based	1		6	P277: ‘Encouraging people to attend their regular appointments whether pregnant or not I think would be the better focus’	2	P451: ‘I am disappointed that now if you are negative for HPV, your swab is not examined anymore. This seems negligent’	
	Time of screen	17	P99: ‘I thought screening had to be delayed after pregnancy – good if it doesn’t’	5	P253: ‘I think 12 weeks postnatal would be better for a cervical screening but understand the appointment with the GP is at 6 weeks’	8	P346: ‘I don’t think there would be big take up for smear tests at 6 weeks. People are still healing, and probably unlikely to want additional intervention so soon after birth’	4
	Self-sampling	17	P227: ‘At home testing would be brilliant’	1	P389: ‘I would do whichever was most effective, urine or smear, and at any stage recommended after pregnancy. I have not had any vaginal births though, and perhaps that has influenced my answers’	1	P235: ‘I think home HPV testing is great for a routine test, but after pregnancy, my brain was so forgetful I would have probably forgot about it’	5
**Self-efficacy**	Speculum-based	2	P70: ‘I feel more confident in the smear test as someone else is doing it than a urine sample’	0		0		1
	Time of screen	3	P212: ‘Having attended routine cervical screening both before and after having a baby, it was much easier after’	3	P327: ‘In theory I actually think this is a great idea. I would say the limitations however at a 6-week check can be the challenges in getting out and timing a longer appointment when you have a newborn. If the baby is crying or needs feeding, etc., this can cause a lot of stress’	15	P57: ‘I couldn’t even comprehend having a smear test after 6 weeks’	
	Self-sampling	0		0		0		

Underlined sections indicate text aligning with construct.

*‘no direction’ = statement of fact, rather than any personal opinion being provided. If participants offered a combination of positive and negative views, this was coded as neutral.

### Qualitative content analysis

Free-text responses were provided by 279 participants. Participants not willing to take part in a cervical screening study during the postnatal period were more likely to have entered a free-text response than those who indicated hypothetical willingness (101/146; 69.2% versus 178/308; 57.8%, respectively; *p* = 0.023; Fisher's exact test). When assessing only those who made comments, the median word count was 32 versus 42 words for those willing and unwilling to take part in a 6-week cervical screening study, respectively (*p* = 0.0123; Mann–Whitney test). Interpretation of these findings therefore should account for this, as free-text responses were more likely to be provided by those with negative feelings about cervical screening or the proposed timing at 6 weeks postnatal.

The affective attitude and burden constructs were assigned the greatest volume of coded data (Table 2); 75% of participants (*n* = 209) had data coded into these constructs. Birth trauma whether physical, psychological, or otherwise was commonly highlighted in women's comments about barriers to postnatal screening (*n* = 24). Fifty (17.92%) participants described feeling worried and fearful of the idea of having speculum screening so close to birth, particularly for those who find the speculum-based test invasive at other times. One participant shared:

P139: *I had a traumatic birth and the thought of having a speculum inserted 6 weeks postpartum would horrify me*. (affective attitude)

Some participants were undecided either because they themselves had experienced both complex and straightforward deliveries (e.g. P325 as quoted above) or because they demonstrated empathy towards others, even if they may not have been affected by these issues personally, such as:

P357: *I had a straightforward vaginal birth, no tears, minimal bleeding etc… but I know of new mums who have had very traumatic births, tearing, heavy blood loss, still bleeding at 6 weeks [postnatal] who would much prefer to be able to do a urine sample than a smear*. (burden)

The importance of cervical screening to individual participants was seen throughout the free-text comments sometimes including a reason, such as previous abnormal results or family history of cervical cancer, but many shared that cervical screening is important, without any further context provided. Participants also commented on earlier screening improving uptake, without specific reasons, although it could be surmised that this was because of perceived convenience (burden):

P161: *I think combining the two appointments is a fantastic idea and I think it will help encourage greater uptake (ethicality)* – the second part of this comment was coded into ethicality demonstrating how the reduction in burden (the practicality of avoiding two appointments) aligns with their value of screening being important.

## Discussion

This mixed-methods study demonstrated that our planned clinical feasibility study of postnatal screening at 6 weeks, timed to coincide with the postnatal baby-check, would be acceptable to two-thirds of women. Four out of five women thought that cervical screening using urine sampling would be acceptable after giving birth. Many felt this would be less burdensome (for themselves or others) after physically and/or psychologically traumatic births. Some were concerned about the accuracy and prioritised this over the burden of conventional cervical screening. More women who had negative attitudes to being offered earlier postnatal cervical screening shared their views, and we will go on to explore the range of opinions, using purposeful sampling in semi-structured interviews to explore the range of opinions in depth and breadth.

From free-text comments, women were aware of several barriers to cervical screening, and although they personally might not prefer to have cervical screening at 6 weeks postnatal, they recognised that they would be more likely to have it performed if offered at a time that they were already attending a primary care appointment. This is in line with what was seen in the YouScreen study, which found that opportunistic screening, using self-sampling methods in non-attenders to the screening program during pre-existing primary care appointments, significantly boosts uptake.^43,44^ In contrast, a smaller increase (12.9%) was observed with the distribution of self-sampling kits to participants’ homes. These data suggest that convenience is a key determinant of engagement in cervical screening programmes and highlight the potential benefits of offering cervical screening during the 6-week postnatal check, particularly as this appointment is in person, which was noted to have the largest increase in screening kits returned (72% compared to 47% with remote consultations).

In 2024, the All-Party Parliamentary Group produced a report on birth trauma, identifying themes contributing to the widespread issue, with around one in three women experiencing a traumatic birth.^45,46^ The implications of this report must be considered when discussing care and interventions postnatally, although a study of pregnant women demonstrated their willingness to be involved in clinical trials.^47^ The themes of both physical and psychological birth traumas were prominent throughout the comment analysis, and consideration and adjustment for this will be incorporated into future study design.

### Strengths

We had a large number of respondents, closing to recruitment once we had saturation of themes within the free-text responses. Through in-depth qualitative analysis of the free-text responses, we gathered more comprehensive data, providing nuanced insight into the breadth and complexity of women's perspectives. The mixed-methods approach allowed triangulation of findings across the quantitative and qualitative approaches.

#### Limitations

The demographics of the participants were reflective of the population in the Southwest of England, where much of the recruitment took place, in terms of age of maternity and ethnicity.^48^ However, as this was a web-based study, we specifically targeted urban mother and baby groups in more diverse areas of cities (including Bristol and Manchester) to address this concern. The web-based design facilitated wider geographical reach and demographically more varied involvement but may have introduced barriers due to lack of literacy and access to devices able to access web-based materials. There were options to contact the team for paper questionnaires and opportunities for translation, but these were not taken up. We intend to assess these aspects in further studies to assess the acceptability of 6-week postnatal screening by approaching people in person following delivery, with a research midwife and access to translation services to assist with questionnaire completion.^49^ The high percentage of participants who had a university degree or post-graduate degree perhaps reflected a high number of respondents who were healthcare staff, as potential participants were more likely to be exposed to information about the study through NHS-based social media and/or share with their friends and colleagues. The free-text comments were in response to an open-ended question about cervical screening, types of tests, and screening after delivery, which provided a wealth of information but with significant variability in question interpretation, and answers were not always specific to postnatal cervical screening.

## Conclusions

Offering cervical screening at the 6-week postnatal check-up is acceptable to many women, and many think this would increase their likelihood of having cervical screening, if offered. Reduced rates of cervical screening may in part be improved by enhancing the accessibility of cervical screening tests and the acceptability of testing methods. This study highlights the importance of offering choice to women, and that a range of options, to make engagement with cervical screening programmes as easy as possible, would likely improve screening rates.

## Supplemental Material

sj-pdf-1-msc-10.1177_09691413251358626 - Supplemental material for Investigating the acceptability of cervical screening, using conventional clinician-taken cervical samples or urine self-sampling, at 6 weeks postnatal: A cross-sectional questionnaire Supplemental material, sj-pdf-1-msc-10.1177_09691413251358626 for Investigating the acceptability of cervical screening, using conventional clinician-taken cervical samples or urine self-sampling, at 6 weeks postnatal: A cross-sectional questionnaire by Victoria Cullimore, Rebecca Newhouse, Holly Baker-Rand, Kim Chu, Sudha Sundar, Emma J Crosbie, Lorna McWilliams and Jo Morrison in Journal of Medical Screening

sj-pdf-2-msc-10.1177_09691413251358626 - Supplemental material for Investigating the acceptability of cervical screening, using conventional clinician-taken cervical samples or urine self-sampling, at 6 weeks postnatal: A cross-sectional questionnaire Supplemental material, sj-pdf-2-msc-10.1177_09691413251358626 for Investigating the acceptability of cervical screening, using conventional clinician-taken cervical samples or urine self-sampling, at 6 weeks postnatal: A cross-sectional questionnaire by Victoria Cullimore, Rebecca Newhouse, Holly Baker-Rand, Kim Chu, Sudha Sundar, Emma J Crosbie, Lorna McWilliams and Jo Morrison in Journal of Medical Screening
