# Supplementary material for: Investigating the acceptability of cervical screening, using conventional clinician-taken cervical samples or urine self-sampling, at 6 weeks postnatal: A cross-sectional questionnaire
Source: J Med Screen. 2025 Jul 21;33(1):48–58. doi: 10.1177/09691413251358626 (PMC12923629; doi:10.1177/09691413251358626)
Supplement: sj-pdf-1-msc-10.1177_09691413251358626 - Supplemental material for Investigating the acceptability of cervical screening, using conventional clinician-taken cervical samples or urine self-sampling, at 6 weeks postnatal: A cross-sectional questionnaire [file sj-pdf-1-msc-10.1177_09691413251358626.pdf]

Attitudes to Postnatal Instead of Normally-Timed Cervical Screening (pre-PINCS-1 study)

Patient information Sheet - Version 1.2 Date: 20/03/2023 IRAS number: 321358

You are being invited to take part in a research study. Before you decide if you would like to take part, it is important for you to understand what it will involve and why the research is being conducted. Please read the following information carefully and take time to decide whether or not you wish to take part. If there is anything that is not clear, or if you would like any further information then please message the research team on PINCSStudy@somersetft.nhs.uk.

Taking part in any research study is entirely your decision, and it is voluntary. Whether you decide to take part, or not, the care that you receive from any medical staff in any healthcare setting will not be affected.

What is this study about?
The purpose of this study is to understand how women who have recently had a baby feel about having cervical screening at their 6-week postnatal check-up. The study will also help us to understand the barriers to screening when you have recently had a baby, and how recently pregnant women feel about the different types of cervical screening tests potentially available in the future (self-tests and cervical smears).

* Required

Participant information section 1/4

Who can take part in this study?
Any woman, aged 24 years or older, who is pregnant or has had a baby within the last 5 years.

Why are we doing this study?
The NHS Cervical Screening Programme (NHS CSP) aims to prevent cancer of the cervix and deaths from this cancer. We know that over half of the women diagnosed with cervical cancer have either never been screened or are out-of-date with their screening. We also know that half of women are not up-to-date with screening by the end of their pregnancy. If more women have screening, more women can be treated to prevent cervical cancer and more lives can be saved.

We have asked groups of new mothers and staff in GP practices what they thought would help women to attend screening. One key idea for change was to move the postnatal smear to coincide with the 6-week postnatal check-up Self-testing was also suggested as a way to improve screening uptake for busy new mums.

With this in mind, we want to understand what stops people attending screening when they have a baby, or small children. To change screening to the 6-week postnatal check-up, we would have to conduct a research study to see if the results of screening were just as accurate at this stage. Before we do that, we want to assess whether women would find it acceptable to have cervical screening 6-weeks postnatal, and what type of screening they would prefer. We are doing this study to collect this information.

Who will conduct the research?
This research is being organized by Dr Holly Baker-Rand, who works at Somerset NHS Foundation Trust. She will be supervised by Ms Lorna McWilliams of the Manchester Centre for Health Psychology, The University of Manchester, and Miss Jo Morrison, Consultant Gynaecological Oncologist at Somerset NHS Foundation Trust.

Participant information section 2/4

Who has reviewed the research?
This research study has been reviewed by an NHS Research Ethics Committee in accordance with local and national guidelines and regulations.

What would I be asked to do if I do agree to take part in the study?
If you decide to take part in this study we will ask you to read an electronic consent form, and acknowledge your agreement to take part.

You will then be transferred to a web-based questionnaire which will take about 15-25 minutes to complete. We will not be collecting any personal identifiable data (e.g. name, address, date of birth) for this study.

However, we are also seeking to explore views in more depth, through an up to 1-hour long interview, in a further study. If this is something you might be willing to contribute to, please provide a contact email address at the end of the survey. This information will not be shared with anyone else.

What happens if I do not take part or if I change my mind?
It is up to you to decide whether or not to take part. If you decide to participate, you will follow a link at the end of this page to a consent form and the web-based questionnaire.

If you do decide to take part you will be asked to show this agreement by acknowledging consent on an electronic consent form. If you decide to take part you are still free to withdraw at any time without giving a reason and without detriment to yourself. However, it will not be possible to remove your data from the project once it has been submitted, as we will not be able to identify your specific data. This does not affect your data protection rights. If you decide not to take part you do not need to do anything further.

For those agreeing to take part in the further in-depth interviews, we will take an audio recording as this is essential to your participation in the study. We will ensure that you are comfortable with the recording process, and you will be free to stop recording at any time. Information about your identity will be removed from this recording and not shared with anyone outside of the research team.

What information will you collect about me?
We will collect information referred to as demographic information. This includes: age, ethnicity, employment status and highest level of education. For those who would be interested in participating in interviews about their views, we will ask for a contact email address.

Participant information section 3/4

What will happen to my information if I decide to take part?
We are collecting and storing this personal identifiable information in accordance with data protection law which protects your rights. These state that we must have a legal basis (specific reason) for collecting your data. For this study, the specific reason is that it is "a public interest task" and "a process necessary for research purposes".

You have a number of rights under data protection law regarding your personal information.

In accordance with data protection law, Somerset NHS Foundation Trust is the Data Controller for this project. This means that we are responsible for making sure your personal information is kept secure, confidential and used only in the way you have been told it will be used. All researchers are trained with this in mind.

Any contact email addresses that are provided will be input in Microsoft Forms. This means it is encrypted at rest and in transit. It is only accessible to fully-trained members of the research team, who will only be able to access this information on secure servers in the Somerset NHS Foundation Trust.

Please also note that individuals from Somerset NHS Foundation Trust or regulatory authorities may need to look at the data collected for this study to make sure the project is being carried out as planned. This may involve looking at identifiable data. All individuals involved in auditing and monitoring the study will have a strict duty of confidentiality to you as a research participant.

How will we use information about you?
We will need to use information from you for this research project.

This information may include your contact details (email address). People will use this information to do the research or to check your records to make sure that the research is being done properly.

People who do not need to know who you are will not be able to see your contact details.

We will keep all information about you safe and secure.

Once we have finished the study, we will keep some of the data so we can check the results. We will write our reports in a way that no-one can work out that you took part in the study.

Participant information section 4/4

What are my choices about how my information is used?
You can stop being part of the study at any time, without giving a reason, but we will keep information about you that we already have.

We need to manage your records in specific ways for the research to be reliable. This means that we won't be able to let you see or change the data we hold about you.

Where can I find out more about how your information is used?
You can find out more about how we use your information

· at www.hra.nhs.uk/information-about-patients/
· by asking one of the research team
· by sending an email to PINCSStudy@somersetft.nhs.uk, or
· by ringing us on 01823 343371.

What should I do if I have a complaint?
If you have a complaint that you wish to direct to members of the research team, please contact:PINCSStudy@somerset-ft.nhs.uk.

The Patient Advisory Liaison Service (PALS) is a confidential NHS service that can provide you with support for any complaints or queries you may have regarding the care you receive as an NHS patient. PALS is unable to provide information about this research study.

If you wish to contact the PALS team, please contact pals@Somersetft.nhs.uk or from the PALS website https://www.somerset-ft.nhs.uk/contact-us/contact-us-and-get-involved/pals/

Alternative contact details for further information

Dr Holly Baker-Rand
GRACE Centre
Musgrove Park Hospital
Taunton
TA1 5DA

Consent form: Pre-PINCS study

1. I confirm that I have read the attached information sheet (version 1.2, 20/03/23) for the above study and have had the opportunity to consider the information, ask questions, and have these answered satisfactorily. I am 24 years old or more, and I am currently pregnant or have delivered a baby in the past 5 years.
2. I understand that my participation in the study is voluntary and that I am free to withdraw at any time without giving a reason and without detriment to myself. I understand that it will not be possible to remove my data once it has been submitted and forms part of the data set. I agree to take part on this basis.
3. I agree to complete an online questionnaire for the research study, as explained to me.
4. I understand that no personal identifying information will be given by me for the purposes of the research, unless I voluntarily choose to provide a contact email address which I agree to be used to contact me about a possible research interview. However, giving my details does not commit me to this second study (there will be a separate patient information sheet and consent form for this interview study).
5. I understand that the data collected during the study may be looked at by individuals from the research team and I give permission for these individuals to access my data.
6. I agree that any data collected may be published in academic reports or journals.
7. I understand that any information collected about me will be processed in accordance with data protection law as explained in the Participant Information Sheet.
8. I agree to participate in this study.

IRAS project ID: 321358. Filing instructions - MS Forms encrypted

1. I have carefully read the consent form and I agree to take part in the study:
   If you do not agree to take part in the study, please leave this page. *

   ◯ Yes

Your views on cervical screening

2. Have you heard of the following?: (tick all that apply) *

- ☐ Cervical (neck of the womb) cancer
- ☐ National cervical screening programme
- ☐ Cervical smear test
- ☐ Human papillomavirus (HPV) self sampling (DIY smear, HPV self-test)

3. When was the last time that you had a cervical smear test? *

- ◯ Within the past 3 years
- ◯ Over 3 years ago
- ◯ Never

4. If you have not attended your smear test ever, or if you have delayed having it, why? *

- ☐ I was not aware of screening
- ☐ I do not have any symptoms
- ☐ I have not had time to attend the appointment
- ☐ I cannot afford the costs linked to attending appointments (travel, parking, time off work)
- ☐ I am scared of the examination and smear test
- ☐ Not applicable (too young or have attended when invited)
- ☐ Other

5. How do you usually get to hospital appointments? *

- ◯ Drive self and use hospital car park
- ◯ Driven by family member/friend
- ◯ Bus
- ◯ Train
- ◯ Taxi
- ◯ Walk
- ◯ Cycle
- ◯ Other

6. How many children have you given birth to? *

- ◯ 0
- ◯ 1
- ◯ 2
- ◯ 3
- ◯ 4
- ◯ 5
- ◯ 6
- ◯ More than 6

7. When did you give birth to your last child? *

- ◯ Currently pregnant
- ◯ Within the past 4 months
- ◯ 4-12 months ago
- ◯ 1-2 year ago
- ◯ 2-3 years ago
- ◯ 3-4 years ago
- ◯ 4-5 years ago

8. Would you be willing to take part in a research study about postnatal cervical screening that involves having a cervical smear 6 weeks after delivery? *

- ◯ Yes
- ◯ No

9. Would you be willing to take part in a research study about postnatal cervical screening that involves providing a urine sample at 6-weeks post-delivery? *

- ◯ Yes
- ◯ No

10. When thinking about having a cervical smear test, please rate your agreement with the following statements: *

	Strongly agree	Agree	Neither agree nor disagree	Disagree	Strongly disagree
I would be more likely to have my cervical smear after pregnancy if it was at the time of my 6-week postnatal check.	◯	◯	◯	◯	◯
I would be more likely to have cervical screening 6-weeks after delivery if I only had to provide a urine sample for testing.	◯	◯	◯	◯	◯
I would prefer to do self-testing by providing a urine sample than have a smear test.	◯	◯	◯	◯	◯
I would prefer to have cervical screening at 6-weeks post-delivery than at 12-weeks post-delivery	◯	◯	◯	◯	◯

11. Please let us know any thoughts that you have about cervical screening, types of tests, or screening after delivery.

12. If you would be willing to participate in an in-depth interview (approx 1 hour) about your views on cervical screening and childbirth, and you are within 6 months of giving birth, please provide a contact email address.

About you

13. Age *

- ◯ 24-29 years old
- ◯ 30-34 years old
- ◯ 35-39 years old
- ◯ 40-44 years old
- ◯ 45 years old and over

14. What is your ethnic group? *

- ◯ Indian
- ◯ Pakistani
- ◯ Bangladeshi
- ◯ Chinese
- ◯ Any other Asian background
- ◯ Caribbean
- ◯ African
- ◯ Any other Black, Black British, or Caribbean background
- ◯ White and Black Caribbean
- ◯ White and Black African
- ◯ White and Asian
- ◯ Any other Mixed or multiple ethnic background
- ◯ English, Welsh, Scottish, Northern Irish or British
- ◯ Irish
- ◯ Gypsy or Irish Traveller
- ◯ Roma
- ◯ Any other White background
- ◯ Arab
- ◯ Any other ethnic group

15. How would you describe your employment status? *

- ◯ Employed or self-employed
- ◯ Unemployed
- ◯ Student
- ◯ Retired
- ◯ Homemaker

16. What is the highest level of education you have completed? *

- ◯ Primary school
- ◯ Secondary school up to 16 years
- ◯ Higher or secondary or further education (A Levels, BTEC etc)
- ◯ College or university
- ◯ Post-graduate degree

This content is neither created nor endorsed by Microsoft. The data you submit will be sent to the form owner.

Microsoft Forms
