# Supplementary material for: Investigating the acceptability of cervical screening, using conventional clinician-taken cervical samples or urine self-sampling, at 6 weeks postnatal: A cross-sectional questionnaire
Source: J Med Screen. 2025 Jul 21;33(1):48–58. doi: 10.1177/09691413251358626 (PMC12923629; doi:10.1177/09691413251358626)
Supplement: sj-pdf-2-msc-10.1177_09691413251358626 - Supplemental material for Investigating the acceptability of cervical screening, using conventional clinician-taken cervical samples or urine self-sampling, at 6 weeks postnatal: A cross-sectional questionnaire [file sj-pdf-2-msc-10.1177_09691413251358626.pdf]

Potential Codes	Meaning	Examples of things that could be coded	Example data
**1.0 Affective Attitude - speculum-based screening**	how individual feels about speculum-based screening	positive or negative emotions, likes and dislikes e.g. relief / anger / fear / disappointed	No concern, part of being a female
**1.1 Affective Attitude - time of screen**	how individual feels about timing of screening	positive or negative emotions, likes and dislikes e.g. relief / anger / fear / disappointed	I would be happy to have my screening at any time. I make sure that I keep up to date with my smears either way.
**1.2 Affective Attitude - self-sampling**	how individual feels about self-sampling for screening	positive or negative emotions, likes and dislikes e.g. relief / anger / fear / disappointed	
**2.0 Burden - speculum-based screening**	the amount of effort (physical, psychological, cognitive) to have speculum-based cervical screening	willingness to have it	
**2.1 Burden - time of screen**	the amount of effort (physical, psychological, cognitive) to have cervical screening at 6 weeks	willingness to have it at 6-weeks	Having had an episiotomy during labour I would not feel comfortable having a smear test at 6 weeks as I was still recovering. (negative)<br><br>I think its a great idea.. I missed my last one because I was pregnant at the time.. and then keep meaning and forgetting to book it now, and when I do I dint have enough time to stay in hold in phone queues. (positive)
**2.2 Burden - self-sampling**	the amount of effort (physical, psychological, cognitive) to use self-sampling tests	willingness to use self-sampling	
**3.0 Perceived Effectiveness - speculum based screening**	whether individual perceives speculum-based screening as achieving its purpose	better to stay as is; benefits/harms	I would rather have a cervical smear as to me I would feel that's more reliable than a urine test
**3.1 Perceived Effectiveness - time of screen**	whether individual perceives timing of cervical screening as linked to achieving its purpose	better to change the time; benefits/harms	
**3.2 Perceived Effectivenes - self-sampling**	whether individual perceives self-sampling screening as achieving its purpose	better to use replace speculum with self-sampling	Not sure is self-testing is as accurate as standard screening?
**4.0 Ethicality - speculum-based screening**	extent to which speculum-based screening fits with person's own values	Safety / Trustworthiness / fairness	That it's super important<br><br>No concern, part of being a female

**4.1 Ethicality - time of screen**	extent to which timing of cervical screening fits with person's own values	Safety / Trustworthiness / fairness	
**4.2 Ethicality - self-sampling**	extent to which self-sampling screening fits with person's own values	Safety / Trustworthiness / fairness	a urine sample much easier as it is <u>far less invasive.</u>
**5.0 Intervention coherence - speculum-based screening**	whether individual understands speculum-based screening (intervention) and how it works	talking about whether it makes sense or not or mention understanding or not	
**5.1 Intervention coherence - time of screen**	whether individual understands timing of screening (intervention) and how it works	talking about whether it makes sense or not or mention understanding or not	I'm not sure I would want a smear that soon after I gave birth, but <u>I understand completely why it would be a good idea to have it done then</u>
**5.2 Intervention coherence - self-sampling**	whether individual understands self-sampling (intervention) and how it works	talking about whether it makes sense or not or mention understanding or not	
**6.1 Opportunity costs - speculum based screening**	what is given up (e.g. time), to take part in speculum-based cervical screening	interferes with other priorities	
**6.2 Opportunity costs - time of screen**	what is given up (e.g. time), to take part in cervical screening at 6-weeks	interferes with other priorities	It would be easier to have it done at 6 weeks <u>to avoid multiple appointments</u>
**6.3 Opportunity costs - self-sampling**	what is given up (e.g. time), to take part in self-sampling	interferes with other priorities	
**7.0 Self-efficacy - speculum based cervical screening**	how confident individual is having speculum-based cervical screening	feeling able to have speculum-based cervical screening	
**7.1 Self-efficacy - time of screen**	how confident individual is having cervical screening 6-weeks post-birth	feeling able to have cervical screening at 6-weeks	
**7.2 Self-efficacy - self-sampling**	how confident individual is doing self-sampling for cervical screening	feeling able to do self-sampling	

Supplementary Table 1 Code book for structured framework analysis – underlined sections indicate text aligning with construct.
